# Comments on the article by A. J. Lecloux (J Nanopart Res (2015) 17:447) regarding the use of volume-specific surface area (VSSA) to classify nanomaterials

**DOI:** 10.1007/s11051-016-3507-x

**Published:** 2016-08-23

**Authors:** Neil Gibson, Hubert Rauscher, Gert Roebben

**Affiliations:** 1 Directorate for Health, Consumers and Reference Materials, Joint Research Centre of the European Commission, Via E. Fermi 2749, 21027 Ispra, VA Italy; 2 Directorate for Health, Consumers and Reference Materials, Joint Research Centre of the European Commission, Retieseweg 111, 2440 Geel, Belgium

**Keywords:** Nanomaterial, Nanoparticles, Nanomaterial definition, Specific surface area, Modelling

## Abstract

In November 2015, an article by A. J. Lecloux was published in this journal (J Nanopart Res, 17:447, 2015). The article focused on the use of volume-specific surface area (VSSA) for the implementation of the European Commission’s recommended definition of “nanomaterial”. In that paper, VSSA values were calculated for polydisperse particulate materials using a particle number-based averaging method which do not agree with earlier results of VSSA simulations of polydisperse materials reported in 2014 by the Joint Research Centre (JRC) of the European Commission (EC). In this contribution, we explain the difference between traditional view of VSSA which was used by the JRC and the proposed model of Lecloux. Through the use of some simple examples for polydisperse materials, it is demonstrated that the latter produces values which neither correspond to the generally accepted definition of VSSA nor relate to the commonly used experimental methods for determining VSSA using gas adsorption. Lecloux’s model therefore does not constitute a basis for practical implementation of the EC’s definition of nanomaterial using gas adsorption techniques.

## Introduction and background

### The EC Recommendation for a definition of “nanomaterial”

In 2011, a definition of the term “nanomaterial” was adopted by the European Commission (EC). The EC recommends the use of this definition to determine whether a material should be considered as a nanomaterial (NM) for legislative and policy purposes in the EU (European Commission 2011). In the following, this definition will be referred to as the “EC NM definition”.

The primary defining criterion in the EC NM definition is the size of the constituent particles: if 50 % or more of the constituent particles in a material—regardless of whether they are unbound, agglomerated or aggregated—have one or more external dimensions between 1 and 100 nm, that material should be classified as a nanomaterial. For all practical purposes, this corresponds to a material consisting of particles for which the median minimum external dimension is 100 nm or less.

The 50 % threshold refers to the number of particles, hence the basic criterion classifying a material as a nanomaterial is particle number based. Nevertheless, the EC NM definition further specifies that, if technically feasible and requested in specific legislation, a material should be considered as a nanomaterial if its volume-specific surface area (VSSA) is greater than 60 m^2^/cm^3^.

### The definition of volume-specific surface area (VSSA)

The VSSA of a material (in m^2^/cm^3^) is derived from the measured values of the more commonly used (mass-)specific surface area (SSA, in m^2^/g). The SSA has been defined by the International Union of Pure and Applied Chemistry (IUPAC) in the following way: “When the area of the interface between two phases is proportional to the mass of one of the phases (e.g. for a solid adsorbent, for an emulsion or for an aerosol), the specific surface area [.…] is defined as the surface area divided by the mass of the relevant phase” (IUPAC 1997). Determination of the SSA of a solid material in air (or in any other dry, gaseous environment) is usually based on whole-sample measurements, e.g. via the Brunauer–Emmett–Teller (BET) method, which uses gas adsorption to measure the total, i.e. inner and outer surface area of a given (particulate) sample. Dividing this by the mass of the measured sample gives the SSA in m^2^/g. To calculate the corresponding VSSA (in m^2^/cm^3^) one multiplies the SSA by the appropriate material skeletal density, i.e. the (average) density in g/cm^3^ of the material of which the particles are made.

### The use of VSSA in the definition of nanomaterials

The above definition and understanding of VSSA is used, for instance, by SCENIHR, the EU Scientific Committee on Emerging and Newly Identified Health Risks (SCENIHR 2010), in the EC NM definition (European Commission 2011) and by Kreyling et al. (2010). The latter authors proposed a definition of NM based entirely on VSSA, and SCENIHR pointed out that the “VSSA is an integral parameter determined from the entire particulate powder material including the whole size range distribution, with all external and/or internal surfaces. It characterises the entire particulate surface area per volume of a solid and/or powder material” (SCENIHR 2010). To indicate whether a material should be considered as a NM, the publications above suggested a threshold of 60 m^2^/cm^3^, based on the fact that a material consisting of monomodal non-porous spherical particles of diameter 100 nm has a VSSA of 60 m^2^/cm^3^. It is clear that for the vast majority of real particulate materials, the VSSA criterion is not directly equivalent to the median minimum dimension criterion. However, under the EC NM definition, the VSSA criterion can only be used to positively identify a nanomaterial and, as we will see, the probability of a false-positive classification based on a VSSA > 60 m^2^/cm^3^ is small, and likely only to be due to particle porosity.

The VSSA of a material consisting of non-spherical particles will usually be different from that of a material that consists only of spherical particles with the same minimum external dimension. However, if the shape of the particles is sufficiently uniform and known, the relationship between minimum external dimension and VSSA can generally be calculated in a relatively straightforward way and the VSSA threshold eventually adapted (Roebben and Rauscher 2014). In practice, the VSSA of a material depends as well on the porosity, shape irregularity and surface roughness of the particles. The VSSA of a particulate material consisting of porous/rough particles is generally higher than that of a material with non-porous/smooth particles with the same external dimensions.

The possibility to exploit the relationship between particle size distribution and VSSA in the implementation of the EC NM definition is of course the reason for JRC to have modelled the VSSA of particulate materials with different particle shape and size distributions (Rauscher and Roebben 2015). The aim was to evaluate whether VSSA values can be found that may serve as additional threshold criteria not only for nanomaterial classification, but also for ‘non-nanomaterial classification’ (i.e. classifying a material as not being a nanomaterial), while being consistent with the original particle number-based criterion in the EC NM definition. Although this would only be feasible with supplementary information (e.g. on particle morphology, porosity or size multimodality), it would in many cases be attractive because there are standardised, established methods to determine the (V)SSA via gas adsorption [e.g. ISO 9277 (2010)] which require considerably less experimental effort and expense than the determination of a number-based particle size distribution.

### Purpose of this paper

In the following, we will discuss two approaches to model the VSSA of a particulate material. The first approach was used in 2014 to make calculations for a report by JRC (Roebben and Rauscher 2014), and in principle calculates the value that would be determined using gas adsorption techniques. The second model was published in 2015 in this journal (Lecloux 2015). The two approaches produce very different VSSA results for polydisperse materials, and in his article, Lecloux argued that the JRC approach was less appropriate regarding implementation of the EC NM definition. Triggered by this statement, we provide in this paper a detailed analysis of both approaches, and use two idealised examples to show that for polydisperse materials the model of Lecloux produces values that (a) do not match the above definition of VSSA and (b) cannot be derived from experimental results using gas adsorption methods.

In the conclusion of his article, Lecloux proposes that the “BET-specific surface area should not be used to calculate the VSSA value because it takes into account the internal porosity of the particles”. Instead, he proposes the use of a method he described in the 1980s (Lecloux 1981; Lecloux et al. 1988) in order to determine the external surface area of the particles. In the discussions and examples in this paper, we will only consider non-porous particles. This streamlines the arguments without limiting the general validity of our conclusions. Our limitation of the model comparison to non-porous particles avoids any reflection about the appropriateness of the different experimental/analytical methods to each model, and renders the BET method entirely suitable for comparisons with both model calculations. In the remainder of this article, we will therefore only refer to the BET method in respect of experimental determination of SSA and VSSA values. In most of the calculations, we will also assume the absence of sintering (loss of surface area at the contacts between particles in a particulate material).

## Considerations regarding particle shape

In his article, in the section entitled “Effect of the particle shape”, Lecloux refers to the JRC report of 2014. The subsection “The JRC approach” starts with several incorrect statements—he states that the JRC “introduces VSSA thresholds that are adapted to the shape of the material… in a simplified way by introducing a shape factor *S*_f_ varying between 1 and 3”. In fact, in the JRC report a shape factor *S*_f_ was never mentioned, although what was proposed and explained in the report is that thresholds of 40 and 20 m^2^/cm^3^ would be more appropriate for needle/rod-shaped particles and platelet/flake-shaped particles, respectively. Lecloux then goes on to specify aspect ratios associated to the non-spherical cases in the “JRC approach”. Such aspect ratios were never defined in the JRC report, and we note here that Lecloux’s referencing in this respect is inaccurate.

**Fig. 1:**
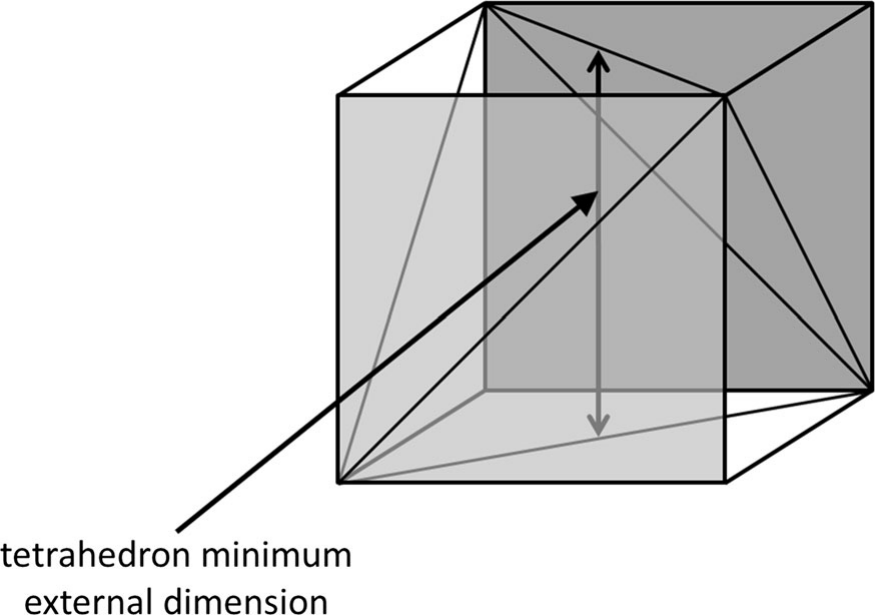
A tetrahedron and its “minimum bounding box”, with the minimum Feret diameter indicated

With regard to different particle shapes, Lecloux acknowledges the fact that different particle size analysis methods result in different particle size values, and that most methods produce an equivalent diameter, rather than a value for the minimum external dimension of a particle. For VSSA measurements, Lecloux correctly deduces the modified VSSA thresholds that would be applicable to rod/fibre-shaped particles or sheet/platelet/flake-shaped particles for a minimum dimension of 100 nm. The same thresholds were deduced in the JRC report (Roebben and Rauscher 2014) to which Lecloux refers. However, in Lecloux’s calculations for tetrahedral, pyramidal and conical particles, he chooses to use a ‘characteristic’ or ‘representative’ dimension, instead of the minimum external dimension (or minimum Feret diameter) relevant for the EC NM definition. For example, the VSSA value of 120 m^2^/cm^3^ for a (regular) tetrahedron of height 100 nm is correct (the height being the distance from the middle of one face to the opposite corner), but such a tetrahedron has a minimum external dimension of ~86.6 nm (the middle of one of the six edges to the middle of the opposite edge, indicated by the arrow in Fig. 1) and therefore does not correspond with the particle size threshold of 100 nm in the EC NM definition. In fact, to have a minimum external dimension of 100 nm, the tetrahedron would need to have a height of ~115.5 nm, and the corresponding VSSA would be ~104 m^2^/cm^3^. The latter value is also shown in Table 1 of Lecloux, but only for the case of the Feret diameter values produced by TEM.

Similarly, most other threshold values calculated by Lecloux for size values deduced from surface area by adsorption measurements or for equivalent sphere measuring methods, are not relevant with respect to the 100 nm size threshold of the EC NM definition. We therefore conclude that Lecloux’s statement that the VSSA threshold values could vary from 20 m^2^/cm^3^ “to 164 m^2^/cm^3^, for pyramidal particles” is not correct when used in the context of the EC NM definition, with the upper limit in fact being 104 m^2^/cm^3^ for tetrahedral particles.

## Calculation of the VSSA of polydisperse materials

Probably, the most puzzling element in the article of Lecloux is his choice to calculate the VSSA of a polydisperse material as a number-weighted average value of the VSSA of the individual particles in that material. The reason for this is not fully clear, but it could be a genuine attempt to find a closer match between the nanomaterial classification approaches based on, on the one hand, the median values of particle number-based particle size distributions and, on the other hand, VSSA values. In this section, we will show that Lecloux’s model actually does not lead to values that match with the accepted definition of VSSA, and that it does not relate directly to values obtained from experimental gas adsorption-based techniques.

### Basic equations

According to the accepted definition of specific surface area (SSA), given by the International Union of Pure and Applied Chemistry (IUPAC 1997), SSA is defined as

1 $${\text{SSA}} = {S \mathord{\left/ {\vphantom {S M}} \right. \kern-0pt} M},$$

where *S* is the total surface area of the sample and *M* is the total mass of the sample. For a material consisting of *n* particles, this equates to

2 $${\text{SSA}} = \frac{{\mathop \sum \nolimits_{i} s_{i} }}{{\mathop \sum \nolimits_{i} m_{i} }}$$

with *s*_*i*_ being the surface area of particle *i* and *m*_*i*_ being the mass of particle *i*. In terms of the SSA of individual particles, SSA_*i*_, this can also be formulated as a mass-weighted average of the SSA_*i*_ values of all individual particles:

3 $${\text{SSA}} = \frac{{\mathop \sum \nolimits_{i} \left( {m_{i} \cdot {\text{SSA}}_{i} } \right)}}{{\mathop \sum \nolimits_{i} m_{i} }} = \frac{{\mathop \sum \nolimits_{i} s_{i} }}{{\mathop \sum \nolimits_{i} m_{i} }}.$$

The VSSA according to SCENIHR (2010), the EC NM definition (European Commission 2011) and Kreyling et al. (2010) is defined as

4 $${\text{VSSA}} = S/V = {\text{SSA}} \times \rho ,$$

where *V* is the total volume of the particles in the sample and *ρ* is the (average) density of the particles. In analogy to the SSA, this can also be formulated as a volume-weighted average of the VSSA_*i*_ of all individual particles:

5 $${\text{VSSA}} = \frac{{\mathop \sum \nolimits_{i} \left( {v_{i} \cdot {\text{VSSA}}_{i} } \right)}}{{\mathop \sum \nolimits_{i} v_{i} }} = \frac{{\mathop \sum \nolimits_{i} s_{i} }}{{\mathop \sum \nolimits_{i} v_{i} }} = \frac{S}{V},$$

where *v*_*i*_ is the volume of particle *i*. Using the above equations and definitions, the SSA can be experimentally determined using the BET method within its range of applicability, for which there are international standards. The calculation of VSSA from the SSA value measured with BET additionally requires the measurement or identification of an appropriate average value for the density of the particulate phase.

The “total VSSA” modelled by Lecloux, which for reasons explained below, we will call *n*VSSA, is based on the following equations, as elucidated in his Discussion section:

6 $$n{\text{VSSA}} = F_{1} \times \frac{{S_{1} }}{{V_{1} }} + F_{2} \times \frac{{S_{2} }}{{V_{2} }} + \cdots + F_{n} \times \frac{{S_{n} }}{{V_{n} }},$$

7 $$n{\text{VSSA}} = F_{1} \times {\text{VSSA}}_{1} + F_{2} \times {\text{VSSA}}_{2} + \cdots + F_{n} \times {\text{VSSA}}_{n},$$

where *F*_*i*_ is the relative frequency of the particles in each of the *n* discrete size classes in the particle size distribution. In fact, and as shown in Eq. (8), this equates to a number-weighted average VSSA of all the individual particles:

8 $$n{\text{VSSA}} = \mathop \sum \limits_{i} F_{i} \cdot {\text{VSSA}}_{i} = \mathop \sum \limits_{i} \left( {\frac{{N_{i} }}{{N_{\text{tot}} }} \cdot {\text{VSSA}}_{i} } \right) = \frac{1}{{N_{\text{tot}} }}\mathop \sum \limits_{i} \left( {N_{i} \cdot {\text{VSSA}}_{i} } \right) = \frac{{\mathop \sum \nolimits_{i} \left( {N_{i} \cdot {\text{VSSA}}_{i} } \right)}}{{\mathop \sum \nolimits_{i} N_{i} }}.$$

We must emphasise here that *n*VSSA does not equate to the commonly accepted definition of VSSA, but is the ‘particle number-based average VSSA’. This particle number-based averaging approach effectively removes the ‘volume-specific’ nature of VSSA, which is why we propose not to name it ‘VSSA’ but ‘*n*VSSA’. One can also note from these equations that *n*VSSA cannot in principle be determined only from a gas adsorption measurement of SSA. Instead, the *n*VSSA calculation requires a priori knowledge of the particle number-based size distribution. This fact will also be illustrated in the examples below.

For earlier publications (Roebben and Rauscher 2014; Rauscher and Roebben 2015), JRC has used VSSA values calculated for model particle systems by applying the basic Eqs. (1–5) shown above. For example, to model a polydisperse sample and calculate a VSSA value that is theoretically equivalent to the widely accepted definition, as well as to what would be measured using the most commonly applied method (gas adsorption), we first determined the sum of the surface areas of the particles in the distribution and then divided this by the sum of the volumes of all particles in the distribution (this being equivalent to dividing by the sum of the masses, and multiplying the result by the material skeletal density, at least in the simple case of materials consisting of particles with a uniform density). This model is expressed by the following equation:

9 $${\text{VSSA}} = \frac{{N_{1} \times S_{1} + N_{2} \times S_{2} + \cdots + N_{n} \times S_{n} }}{{N_{1} \times V_{1} + N_{2} \times V_{2} + \cdots + N_{n} \times V_{n} }} = \frac{{S_{\text{total}} }}{{V_{\text{total}} }} = \frac{{S_{\text{total}} }}{{M_{\text{total}} }} \times \rho,$$

where *N*_*i*_ is the number of particles in each of the *n* size classes. Dividing above and below by *N*_total_ means we can replace *N*_*i*_ with number frequencies *F*_*i*_ in both numerator and denominator. The last term is valid if the sample consists of a single material (i.e. all particles have the same density). This model and its implications for the use of VSSA as a potential defining criterion for nanomaterials have been used in (Roebben and Rauscher 2014) to calculate VSSA values for model particle size distributions in order to compare theoretical VSSA values with the existing VSSA threshold value specified in the EC NM definition (60 m^2^/cm^3^) to indicate that a material is a nanomaterial.

In the following sections, we will use two simple idealised examples to show that the alternative version of VSSA proposed by Lecloux (i.e. *n*VSSA) does not relate to the widely accepted definition of VSSA, and to illustrate why it does not provide a basis for practical implementation of the EC NM definition using gas adsorption methods.

### Example 1: bimodal distributions

In his article, Lecloux makes some considerations on bimodal particle size distributions based on his *n*VSSA calculations. He states for example: “As a results, it appears that any combination of at least 50 % in number of a lognormal distribution characterised by a *μ* value less than 50 nm with any other lognormal distribution characterised by a *μ* value higher than 100 nm will lead to a VSSA value higher than the threshold, even if there is less than 50 % number of nanoparticle in the total sample”. By “*μ* value” Lecloux intends the median of the distribution (though in fact the median of a lognormal distribution is given by e^*μ*^, as earlier noted by Lecloux in his article). In order to be sure of our interpretation, we have reproduced some of the values of Table 8 of the Lecloux article using lognormal distributions with median values as specified in the first column and *σ*_*y*_ values as specified in the first row.

The *n*VSSA values produced with the model of Lecloux will indeed lead to the results reported in his Table 8, and to his above conclusion, since the VSSA of a 50-nm-diameter spherical particle is 120 m^2^/cm^3^ and that of smaller particles is greater than this value, while the VSSA of particles larger than 100 nm will range from 60 m^2^/cm^3^ to nearly zero for very large particles. Thus for two narrow lognormal distributions, if the number of particles in the distribution with the median value <50 nm is at least 50 %, then the calculation of Lecloux leads to *n*VSSA > 60 m^2^/cm^3^. We have also checked that this is the case for broadened lognormal distributions. The question, however, is whether the same is true when using traditional VSSA values.

Let us therefore examine a hypothetical case where Lecloux claims the VSSA should be higher than the threshold: imagine a bimodal sample with 2 × 10^13^ non-porous, spherical nanoparticles with a median diameter of 10 nm, combined with 10^13^ particles of the same material with a median diameter of 500 nm. For the sake of argument, we will assume that the two individual distributions are lognormal but very narrow. The *n*VSSA value is a little over 400 m^2^/cm^3^. However, in terms of the VSSA as intended by Kreyling et al. (2010), SCENIHR (2010), and the EC NM definition (European Commission 2011), the conclusion is different. The surface area of all the larger 500 nm particles combined is 7.85 m^2^, and their combined volume is 0.6545 cm^3^. The VSSA of these particles alone is therefore 12 m^2^/cm^3^. This corresponds with the expected VSSA value (in m^2^/cm^3^) which can be calculated for monodisperse spherical particles by dividing 6000 by the diameter (in nanometers). The surface area of all the smaller 10 nm particles combined is 0.00628 m^2^, and their combined volume is 0.00001047 cm^3^. If one adds the 2 × 10^13^ smaller particles to the 10^13^ larger ones, adding less than 0.1 % to the surface area of the entire sample and a negligible amount to the total volume (or mass), the measured VSSA value will hardly change, remaining within experimental error at 12 m^2^/cm^3^.

We note that Lecloux states in his conclusions that his approach should be used only for monomodal particle size distributions. However, this example is still relevant, firstly with respect to Lecloux’s statements on bimodal materials, but secondly and more importantly, to underline the fact that the *n*VSSA values derived from the model of Lecloux are not equivalent to VSSA values in the ‘classical sense’. It also illustrates that to determine *n*VSSA values an in-depth knowledge of the particle size distribution is essential and also that gas adsorption measurements produce values that are unrelated to *n*VSSA in such cases.

### Example 2: distribution broadening

In his article, Lecloux also states that “due to its overprotective character, the VSSA criteria can be a good tool to identify “non-nano” materials. As soon as the VSSA value is below the threshold, it is a clear indication that the powder is not a nanomaterial”.

This statement is based on the belief that broadening of a monomodal distribution, while maintaining a constant median value, will increase rather than decrease the VSSA (Lecloux 2015). Let us examine this with a simple example. We know that a monodisperse sample of spherical particles of 100 nm diameter has a VSSA of 60 m^2^/cm^3^. Now assume a very simple broadened version of this distribution—for ease of calculation, we take nine spherical particles: one of 80 nm, two of 90 nm, three of 100 nm, two of 110 nm and one of 120 nm.

The total surface area of this ensemble (in nm^2^) is 20106 + 2×(25447) + 3×(31416) + 2×(38013) + 45239 = 286513 nm^2^ and its total volume (in nm^3^) is 268082 + 2×(381703) + 3×(523598) + 2×(696909) + 904778 = 4900878 nm^3^.

The VSSA that would be determined by BET is therefore 0.05846 nm^2^/nm^3^ = 58.46 m^2^/cm^3^. This is less than the threshold, illustrating that Lecloux’s suggestion that broadening of a distribution while maintaining a median value of 100 nm will increase the measured VSSA, is incorrect. The conclusion by Lecloux that VSSA is overprotective therefore has to be rejected. It is true that the theoretical value of *n*VSSA would indeed increase for this distribution, but measurement by gas adsorption would in principle not match the calculated *n*VSSA value, since there is no other physical surface available (in addition to that calculated above for the nine particles) to bring the experimentally determined VSSA value above the threshold. This again illustrates that gas adsorption methods cannot be used to determine Lecloux’s *n*VSSA and that a thorough knowledge of the particle number-based size distribution is required for its evaluation.

In order to complete the picture concerning distribution broadening, we draw attention to Figs. 9 and 10 of Lecloux’s article. The blue lines in those figures are calculated according to the formula for *n*VSSA. The orange lines are calculated with what Lecloux refers to as the “JRC model”, and which we have demonstrated is equivalent to what would be determined using gas adsorption techniques (for example the BET method). It is interesting to note how much the measured VSSA (for a median particle size value of 100 nm) will decrease below the threshold for significantly broadened, but monomodal, particle size distributions, especially lognormal distributions, with a 50 % reduction reported in Lecloux’s Fig. 10 for a standard deviation of 0.6. This serves to underline the fact that distribution broadening has a significant reducing effect on VSSA and therefore an experimentally determined VSSA value lower than the 60 m^2^/cm^3^ threshold cannot be used as a simple indication that a material is a “non-nanomaterial”.

## Conclusions

In the article of Lecloux (2015), one can find a number of inaccurate references to other work, in particular that of JRC (Roebben and Rauscher 2014). In contrast with the claims made by Lecloux in his article regarding the earlier work of the JRC, the arguments and examples presented above illustrate the validity of the approach used by JRC to calculate VSSA according to its generally accepted definition and as measured by the most common experimental method (gas adsorption). The differences reported by Lecloux between his modelling results and those of JRC are caused by the fact that his model does not produce VSSA values, but particle number-based (arithmetic) average VSSA values, which we have termed *n*VSSA values.

While Lecloux’s proposal to use *n*VSSA instead of VSSA for implementing the EC NM definition may be a genuine attempt at creating a more suitable measurand, his apparent belief that this can be reliably linked to gas adsorption measurements is fundamentally flawed, and has led Lecloux to draw a number of misleading conclusions. In particular, at various points in his article, Lecloux labelled the VSSA criterion as “overprotective”, meaning that many materials would wrongly be classified as nanomaterials using the VSSA criterion, and that, as he stated in his conclusions, “….as soon as the VSSA is below the threshold, the sample can be considered as a non-nanomaterial”. We have shown that, with respect to the currently recommended EC NM definition, the opposite is the case and that, if the VSSA value is below the threshold, the sample should not necessarily be considered as a non-nanomaterial.

We point out that the *n*VSSA calculations require a priori knowledge of the number-based particle size distribution. Therefore, as opposed to VSSA calculations based on gas adsorption measurements, the *n*VSSA approach proposed by Lecloux is not an alternative for the more complex number-based particle size distribution measurements. We stress that the VSSA thresholds in the current EC NM definition assume a particle volume-weighted approach, and that only by modelling VSSA in this way can thresholds be derived that may be usable as practical criteria for implementing the EC NM definition.
